# Development and Application of a Synthetically-Derived Lead Biosensor Construct for Use in Gram-Negative Bacteria

**DOI:** 10.3390/s16122174

**Published:** 2016-12-18

**Authors:** Lara Bereza-Malcolm, Sanja Aracic, Ashley E. Franks

**Affiliations:** Applied and Environmental Microbiology Laboratory, Department of Physiology, Anatomy and Microbiology, La Trobe University, Melbourne, Victoria 3086, Australia; ltbereza-malcolm@students.latrobe.edu.au (L.B.-M.); S.Aracic@latrobe.edu.au (S.A.)

**Keywords:** whole cell biosensors, lead, synthetic biology, environmental monitoring

## Abstract

The use of lead in manufacturing has decreased significantly over the last few decades. However, previous widespread use of lead-containing products and their incorrect disposal has resulted in environmental contamination. Accumulation of harmful quantities of lead pose a threat to all living organisms, through inhalation, ingestion, or direct contact, resulting in lead poisoning. This study utilized synthetic biology principles to develop plasmid-based whole-cell bacterial biosensors for detection of lead. The genetic element of the lead biosensor construct consists of *pbrR*, which encodes the regulatory protein, together with its divergent promoter region and a promoterless *gfp*. GFP expression is controlled by PbrR in response to the presence of lead. The lead biosensor genetic element was cloned onto a low-copy number broad host range plasmid, which can stably exist in a range of laboratory and environmental isolates, including *Pseudomonas*, *Shewanella*, and *Enterobacter*. The biosensors constructed were found to be sensitive, rapid, and specific and could, as such, serve as monitoring tools for lead-contaminated water.

## 1. Introduction

Lead is a naturally-occurring heavy metal constituting 10–30 mg·kg^−1^ of Earth’s crust [1]. Due to its unique characteristics it has been used extensively in the manufacturing of a wide range of products including: lead-acid batteries, paint, plumbing pipes, leaded gasoline, and ammunition. It is soft, highly malleable, dense, ductile, resistant to corrosion, non-degradable, and able to react with organic chemicals. Lead has only started being phased out of use in manufacturing over the last few decades even though its toxicity has been documented for hundreds of years [2]. Lead poisoning can occur as a consequence of ingesting, inhaling, or via direct contact with lead-containing compounds [1]. Once it enters the body it is able to cross the blood-brain barrier and damage the central and peripheral nervous systems [3]. This is through the mimicking of other essential metals, such as calcium, iron, and zinc, thus interfering with enzymes’ abilities to catalyze reactions [4,5]. Damage to the central nervous system is particularly prevalent in children as lead interferes with synapse formation, neurochemical development, and the organization of ion channels, leading to reduced cognitive ability [6]. The World Health Organization (WHO) 2011 guidelines recommend a provisional guideline value of 10 µg·L^−1^ for lead in drinking water [7]. 

Detection of heavy metals in the environment requires use of analytical techniques, including atomic absorption spectrometry and inductively-coupled plasma-atomic emission spectrometry [8]. These methods of analysis require transport of the potentially contaminated sample to a laboratory and specialist knowledge, thus resulting in a time delay between sample collection and contaminant identification. There is an increasing demand for complementary and alternative in situ detection methods, which can also determine the amount of bioavailable heavy metal [9]. The bioavailability of lead depends on multiple factors; the physicochemical properties of the lead-containing compound, the environmental matrix, as well as the biological recipient and its physiology [10].

Bacteria respond rapidly to low concentrations of heavy metals in comparison to other members of soil biota; therefore, they are of increasing importance for use in toxicity tests of groundwater, soil, and sediments [11,12]. Lead resistance has been reported in both Gram-negative and Gram-positive bacteria [13]. These mechanisms are often coupled with reduction and efflux systems of other heavy metals. For example, lead resistance has been reported to be coupled with cadmium [14] and mercury [15] resistance. The first lead-specific resistance locus (*pbrTRABCD*) was identified on a mobile genetic element, the pMOL30 plasmid of *Ralstonia metallidurans* CH34 (later re-named *Cupriavidus metallidurans* CH34) [16]. The locus encodes proteins that are involved in lead uptake (PbrT), efflux (PbrA; a P-type ATPase, PbrB; a phosphatase; and PbrC, a prolipoproprotein signal peptidase), and sequestration (PbrD). The *pbrABCD* operon is regulated by a transcriptional regulator, PbrR, belonging to the MerR family of metal ion-sensing regulatory proteins. Similar lead loci (*pbrRABC*) have also been identified on other plasmids, such as the pLVPK virulence plasmid from *Klebsiella pneumoniae* [17] and the pEC-IMPQ plasmid from *Enterobacter cloacae* [18].

The understanding of resistance mechanisms has enabled synthetic biologists to re-design the system to enable expression of a detectable and measurable output signal, which is directly proportional to the concentration of a specific analyte [9]. A biosensor genetic element containing a regulatory gene, together with its promoter region and a reporter gene, can be introduced into microbial species either on a plasmid or directly into the chromosome producing a microbial biosensor. Previous plasmid-based lead microbial biosensors have utilized the genetic element from the cadmium resistance mechanism with a luminescent output [19,20]. Not surprisingly, these lead biosensors also responded to low levels of cadmium (and zinc) due to the genetic element used. A chromosomal-based lead microbial biosensor utilized *pbrR* and its native divergent promoter region, P*_pbr_*, to allow expression of the luciferase operon (*luxCDABE*). However, in addition to lead, the biosensor was also responsive to mercury, cadmium, and zinc [21]. A range of microorganisms have been utilized as lead microbial biosensors, including Gram-negative *Alcaligenes eutrophus* CH34 [22], *Escherichia coli* and *Pseudomonas fluorescens* [21], and Gram-positive *Staphylococcus aureus* and *Bacillus subtilis* [19,20].

The aim of this study was to develop lead microbial biosensors that could be used for ecotoxicological assessment of lead-polluted environmental samples. Furthermore, we aimed to increase knowledge of the use of *pbrR* and its divergent promoter region in lead biosensor constructs, and their potential for differential response in various Gram-negative bacteria. A plasmid-based, lead biosensor construct was developed and transferred into *Pseudomonas aeruginosa* PAO1, *Shewanella oneidensis* MR-1 and two wild-type *Enterobacter* sp. isolated from heavy metal contaminated soil. The latter two were selected because of their environmental relevance and bioremediation abilities [23]. The biosensor construct contained the *pbrR* gene and its native divergent promoter region (P*_pbr_*) from the pLVPK plasmid of *K. pneumoniae* CG43, in addition to a promoterless *gfp* gene. Quantitative analyses were utilized to determine the limit of detection, the response time and the specificity to lead nitrate (Pb(II)) of the five microbial biosensors. To determine the feasibility of the microbial biosensors for in situ studies, two spiked environmental samples, tap water and groundwater, were tested. Previously, lead biosensors have only been tested in response to lead in soil samples [20], therefore, to the best of the authors’ knowledge this is the first study to develop lead-specific biosensors with a fluorescent output for testing of lead-contaminated water samples.

## 2. Materials and Methods

### 2.1. Bacterial Strains and Culture Conditions

The bacterial strains and plasmids used in this study are listed in Table 1. *E. coli* DH5α and *P. aeruginosa* PAO1 were grown at 37 °C, while *S. oneidensis* MR-1, *Enterobacter* sp. NCR3 and *Enterobacter* sp. LCR17 were grown at 28 °C. All strains were cultured on nutrient agar (NA) and in nutrient yeast broth (NYB) supplemented with antimicrobial agents, as necessary. NA contains 3.5% blood agar base (*w*/*v*) (1.5% (*w*/*v*) agar, 1% (*w*/*v*) ‘Lab-Lemco’ powder, 1% (*w*/*v*) peptone, 0.5% (*w*/*v*) NaCl) and 0.5% (*w*/*v*) yeast extract. NYB contains 2.5% (*w*/*v*) nutrient broth and 0.5% (*w*/*v*) yeast extract.

### 2.2. Antimicrobial Agents

Stocks of antibiotics and heavy metals were prepared with sterile distilled water (dH_2_O) at 10^4^ µg·mL^−1^. Antimicrobial agents were freshly diluted in dH_2_O to appropriate concentrations as necessary. Selection for the transfer of pCR2.1^®^-TOPO^®^ TA cloning vector (Invitrogen^TM^, Life Technologies, Carlsbad, CA, USA) and the broad-host range plasmid, pBBR1MCS-5, was achieved on NA containing ampicillin (100 µg·mL^−1^) and gentamicin sulfate (10 µg·mL^−1^), respectively. A nalidixic acid resistant (7 µg·mL^−1^) laboratory strain *E. coli* DH5α was used for construction of the lead biosensor construct. Heavy metals used for testing of the biosensors included: sodium arsenite (As(III)), cadmium chloride (Cd(II)), copper chloride (Cu(II)), chromium oxide (Cr(VI)), lead nitrate (Pb(II)), mercury chloride (Hg(II)) and zinc chloride (Zn(II)). Antibiotics (ampicillin, gentamicin sulphate and nalidixic acid) were stored at 4 °C and heavy metal compounds were stored at room temperature. All antimicrobial agents were obtained from Sigma-Aldrich Pty Ltd. (St. Louis, MO, USA). 

### 2.3. Construction of the Plasmid-Based Lead Biosensor Construct

The genetic element containing the *pbrR* gene (435 bp) and the divergent promoter region (P*_pbr_*; 85 bp) that it regulates from *K. pneumoniae* CG43 plasmid pLVPK (NCBI Accession No. AY378100; coordinates: 155782–56301; 520 bp) and a promoterless *gfp* gene (*gfpmut3b*; 720 bp; iGEM biobrick BBa_E0040), was synthesized by Integrated DNA Technologies. The synthesized fragment (1240 bp) was cloned into pCR^®^ 2.1-TOPO^®^ TA (Invitrogen^TM^, Life Technologies, Carlsbad, CA, USA) (3931 bp) and subsequently subcloned into the *Eco*RI (Promega (Madison, WI, USA)) site of pBBR1MCS-5 (4768 bp). Blue-white screening was employed to detect the vector containing the recombinant DNA (i.e., pBB*pbrRgfp*). Isolated plasmid DNA was subjected to *Eco*RI restriction digestion at 37 °C for 1 h and gel electrophoresis to visualise the release of the correct sized fragment. Both pTOPO*pbrRgfp* and pBB*pbrRgfp* were Sanger sequenced at the Australian Genome Research Facility to confirm the genetic element cloned.

### 2.4. Conjugation Procedure

Aliquots (1 mL) of exponential phase donor (*E. coli* WM3064(pBB*pbrRgfp*)) and recipient (either *P. aeruginosa* PAO1, *S. oneidensis* MR-1, *Enterobacter* sp. NCR3 or *Enterobacter* sp. LCR17) NYB-cultures (~10^9^ cfu·mL^−1^) were mixed and filtered through a nitrocellulose membrane filter (0.45 µm pore size; 25 mm diametre; Millipore (Cork, County Cork, Ireland). The filter with the bacteria facing uppermost was then placed onto the surface of a pre-warmed NA plate. After incubation (2–3 h for *P. aeruginosa* PAO1 or 4–5 h for *S. oneidensis* MR-1 and *Enterobacter* spp.) the bacteria were resuspended in 2 mL NYB, serially diluted (ten-fold in saline) and aliquots (100 µL) plated onto selective NA containing gentamicin sulfate at 10 µg·mL^−1^, allowed to dry and incubated under appropriate conditions. Aliquots (100 µL) of donor and recipient cultures were plated separately onto the same selective medium to serve as negative controls.

### 2.5. Fluorescence Microscopy

Exponential-phase NYB-cultures (OD_600 nm_ = 0.6–0.8) of lead biosensors (*E. coli* DH5α(pBB*pbrRgfp*), *P. aeruginosa* PAO1(pBB*pbrRgfp*), *S. oneidensis* MR-1(pBB*pbrRgfp*), *Enterobacter* sp. NCR3(pBB*pbrRgfp*) and *Enterobacter* sp. LCR17(pBB*pbrRgfp*)) containing antibiotics were induced with a concentration of 100 µg·mL^−1^ Pb(II) for 24 h to allow expression of GFP. An identical NYB-culture was left uninduced and also incubated for 24 h to serve as a negative control. After 24 h, a drop of culture (10 µL) was placed onto a glass slide and covered with a cover slip. The cover slip was firmly pressed onto the slide and sealed using nail polish. Samples were viewed using a Nikon Eclipse Ti-E inverted microscope. Phase contrast was used to view the bacterial cells, and the GFP filter (excitation 485 nm and emission 535 nm) was used to visualise the fluorescent cells at ×1000 magnification with oil immersion. 

### 2.6. Fluorescence Assays

Pb(II) at varying concentrations (or other heavy metals in the case of specificity assays) was added to exponential phase NYB-cultures of lead biosensors and incubated for an appropriate period of time with shaking in the dark. For environmental assays, 900 µL of exponential phase NYB-cultures of lead biosensors were added to either dH_2_O, tap water or groundwater (in 100 µL aliquots) spiked with either 0, 2, or 5 µg·mL^−1^ Pb(II). The groundwater and tap water used were collected from a lake system and the laboratory tap in Victoria, Australia. Following up to 4 h of incubation, the cells were pelleted and washed three times in dH_2_O. For time detection assays cells were pelleted and washed at consecutive time intervals after induction. After washing, cells were aliquoted (200 µL) into black 96-well plates with clear flat bottom which allowed successive measurement of the relative fluorescence units (RFU) and optical density (OD_600 nm_) of the bacterial cells. A Clariostar plate reader was used for measurements of RFU (with the excitation and emission values set at 485 nm and 535 nm, respectively) and OD_600 nm_.

For each assay, three independent biological replicates were performed in triplicate, unless otherwise stated. The mean RFU and OD_600 nm_ of triplicate samples was calculated to obtain the mean RFU per OD_600 nm_ of each biological replicate. Standard error was determined through the use of the students *t*-test (*p* < 0.05), whereby the mean RFU per OD_600 nm_ of the three biological replicates of the uninduced sample was compared to the other samples being tested. The *p* value was calculated by comparing the variability between the mean RFU per OD_600 nm_ of the lowest concentration used during the fluorescence assay (i.e., 0 µg·mL^−1^ Pb(II)) or from the earliest time point during time assays (0 min) to that of other concentrations used or time points, respectively. In each fluorescence assay the mean RFU per OD_600 nm_ of the three biological replicates of the uninduced sample was subtracted from that of the induced samples (i.e., RFU(induced)/OD_600 nm_(induced) − RFU(uninduced)/OD_600 nm_(uninduced)). 

## 3. Results

### 3.1. Construction of the Plasmid-Based Lead Biosensor Construct

**Figure 1:**
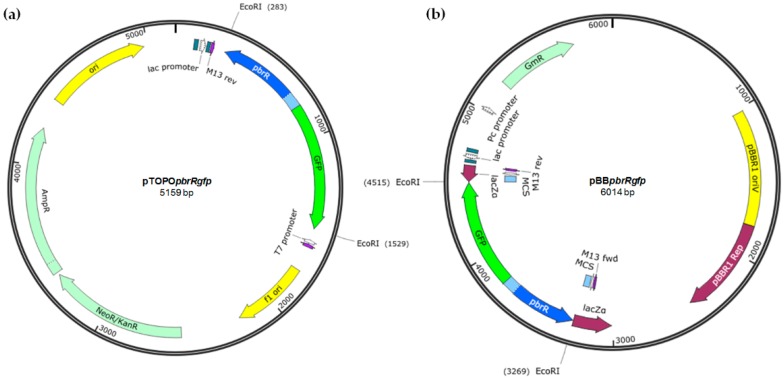
Schematic map of the lead biosensor constructs; (**a**) pTOPO*pbrRgfp* and (**b**) pBB*pbrRgfp*. The *pbrR* gene (435 bp; blue), the divergent promoter region P*_pbr_* (85 bp; pale blue) and a promoterless *gfp* gene (720 bp; green) were synthesized and cloned into pCR^®^ 2.1-TOPO^®^ TA (3931 bp). The lead biosensor genetic element (1240 bp) was then subcloned into the *Eco*RI site of the broad host range pBBR1MCS-5 (4768 bp) plasmid for transfer into other Gram-negative bacteria.

The synthesized biosensor genetic element containing *pbrR*, P*_pbr_* and a promoterless *gfp* gene (1240 bp) was cloned into the pCR2.1^®^-TOPO^®^ TA cloning vector (Invitrogen^TM^, Life Technologies, Carlsbad, CA, USA) (Figure 1a) and subsequently subcloned into the *Eco*RI site of the low-copy number broad host range plasmid pBBR1MCS-5 (4768 bp; Figure 1b). The resulting plasmid (pBB*pbrRgfp*) was transformed into *E. coli* WM3064 and transferred via conjugation to *P. aeruginosa* PAO, *S. oneidensis* MR-1 and *Enterobacter* sp. NCR3 and *Enterobacter* sp. LCR17. *E. coli* DH5α was used for cloning the genetic element and was, therefore, also included in the assessment. 

### 3.2. Qualitative Assessment of the Escherichia coli Lead Biosensor

**Figure 2:**
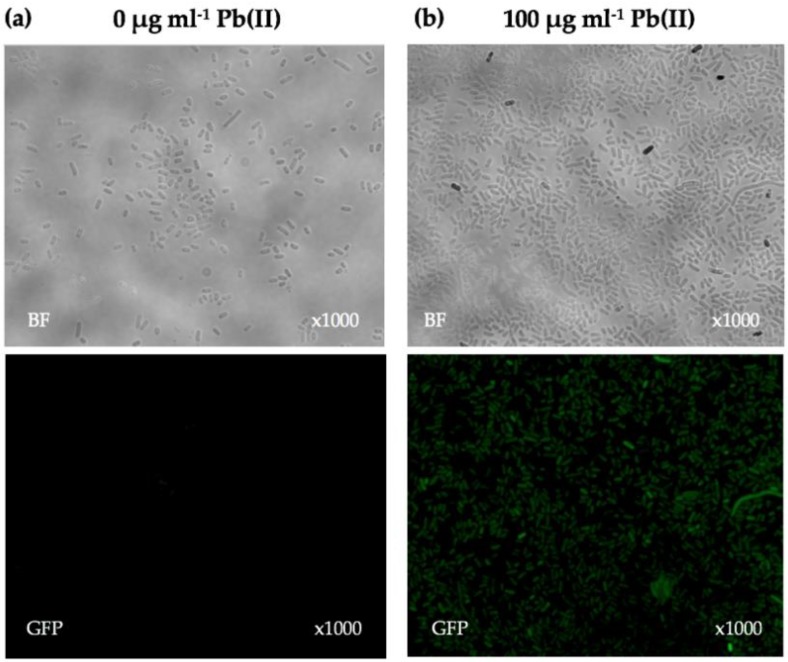
Bright-field (**top**) and fluorescence (**bottom**) microscopy images of *E. coli* DH5α(pBB*pbrRgfp*) NYB-cultures (**a**) uninduced; and (**b**) induced, with 100 µg·mL^−1^ Pb(II) for 24 h at 37 °C.

Initial qualitative analysis of *E. coli* DH5α(pBB*pbrRgfp*) was conducted using fluorescence microscopy to ensure functionality of the pBB*pbrRgfp* construct prior to its transfer into other Gram-negative bacteria. Pb(II) (100 µg·mL^−1^) was added to an exponential phase *E. coli* DH5α(pBB*pbrRgfp*) NYB-culture and incubated for 24 h. An identical exponential phase NYB-culture, lacking Pb(II), served as a negative control. No fluorescent cells were observed in the uninduced NYB-culture (Figure 2a). In contrast, fluorescent cells were observed in the induced NYB-culture, thus confirming the functionality of the biosensor to respond to Pb(II) (Figure 2b). Differences in cell numbers observed in brightfield images of Figure 2a,b are due to sample preparation (live cell cultures being pressed onto a glass slide resulting in an uneven distribution of cells) and not due to treatment (exposure to 0 or 100 µg·mL^−1^ Pb(II)), as OD_600 nm_ was comparable between the samples (~1.2 OD_600 nm_ for both samples). It was also noted that the fluorescent cells in the induced NYB-culture were not fluorescing at the same intensity.

### 3.3. Quantitative Assessment of Lead Biosensors

**Figure 3:**
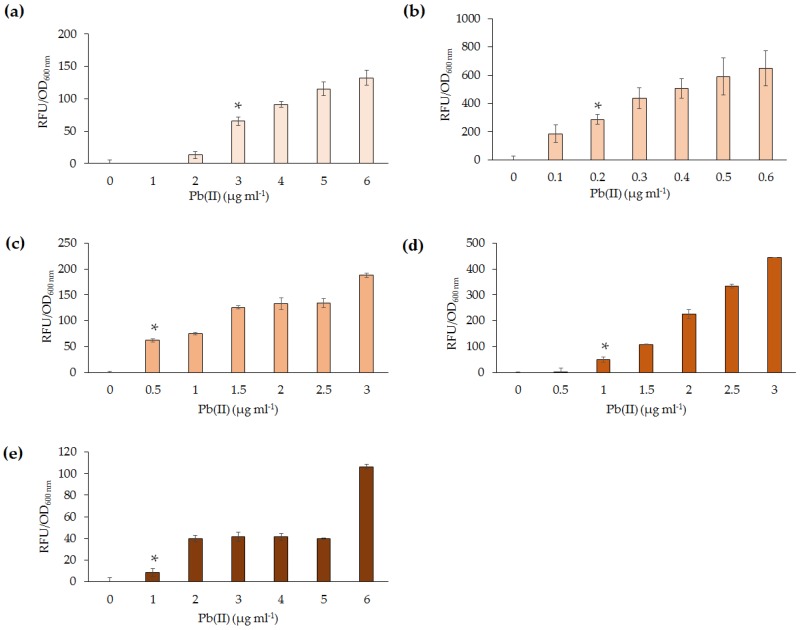
The limit of detection was determined to range between 0.2 and 2 µg·mL^−1^ Pb(II) for the developed lead biosensors (* *p* < 0.05). The *p*-value indicates a significant increase in RFU per OD_600 nm_, in comparison to the same biosensor exposed to 0 µg·mL^−1^ Pb(II). All concentrations after the lowest detection limit are significant in comparison to 0 µg·mL^−1^ Pb(II). (**a**) *E. coli* DH5α(pBB*pbrRgfp*); (**b**) *P. aeruginosa* PAO1(pBB*pbrRgfp*); (**c**) *S. oneidensis* MR-1(pBB*pbrRgfp*); (**d**) *Enterobacter* sp. NCR3(pBB*pbrRgfp*); and (**e**) *Enterobacter* sp. LCR17(pBB*pbrRgfp*). Note: For *S. oneidensis* MR-1(pBB*pbrRgfp*) and *Enterobacter* sp. LCR17(pBB*pbrRgfp*) only one biological replicate is shown.

The limit of detection was determined by exposing the five lead microbial biosensors to varying concentrations (0–6 µg·mL^−1^) of Pb(II) for 4 h at their optimal growth temperatures. It was noted that there were several differences in sensitivity between the five biosensors (Figure 3). *P. aeruginosa* PAO1(pBB*pbrRgfp*) was found to respond to the lowest concentration of Pb(II) at 0.2 µg·mL^−1^ (Figure 3b). At the limit of detection, this biosensor exhibited the highest RFU/OD of the five biosensors tested (~400 RFU/OD versus <100 RFU/OD; Figure 3). The *Enterobacter* spp. wild-type isolates detected 1 µg·mL^−1^ Pb(II) (Figure 3d,e) while *S. oneidensis* MR-1(pBB*pbrRgfp*) detected 0.5 µg·mL^−1^ Pb(II) (Figure 3c).

**Figure 4:**
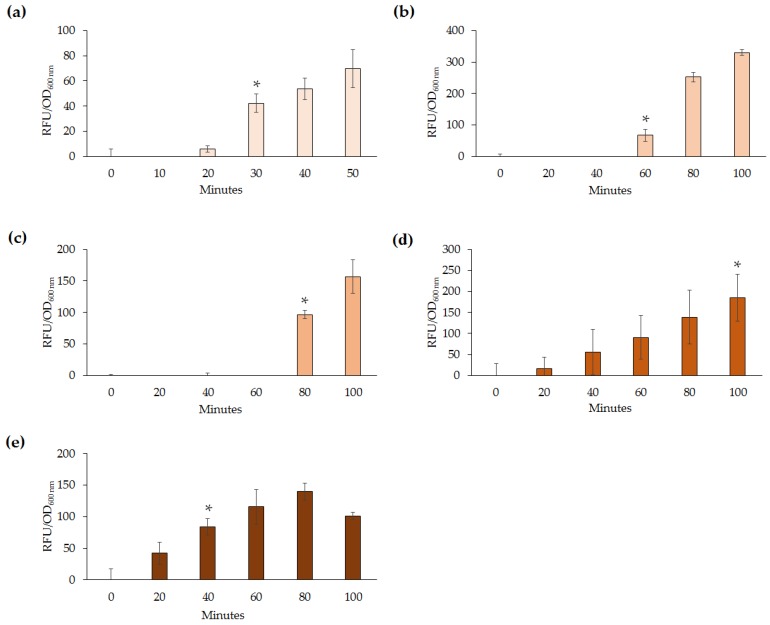
The time taken until a detectable response was determined for the five lead biosensors was found to range between 30 and 100 min after the addition of varying concentrations of Pb(II) (* *p* < 0.05). The *p*-value indicates the earliest significant increase in RFU per OD_600 nm_, in comparison to the time point 0 after induction with Pb(II). (**a**) *E. coli* DH5α(pBB*pbrRgfp*) (5 µg·mL^−1^); (**b**) *P. aeruginosa* PAO1(pBB*pbrRgfp*) (0.2 µg·mL^−1^); (**c**) *S. oneidensis* MR-1(pBB*pbrRgfp*) (5 µg·mL^−1^); (**d**) *Enterobacter* sp. NCR3(pBB*pbrRgfp*) (2 µg·mL^−1^); and (**e**) *Enterobacter* sp. LCR17(pBB*pbrRgfp*) (5 µg·mL^−1^). Note: For *S. oneidensis* MR-1(pBB*pbrRgfp*) only one biological replicate is shown. For *Enterobacter* sp. LCR17(pBB*pbrRgfp*) two biological replicates are shown.

For the developed lead biosensors to complement current analytical techniques it is essential that they are able to rapidly detect Pb(II). As such the five lead microbial biosensors constructed were tested for their ability to detect varying concentrations of Pb(II) over different time intervals. The RFU were measured every 10–20 min intervals until a statistically significant increase from time point 0 was established. All five microbial biosensors were found to respond within 30–100 min after induction with Pb(II) (Figure 4).

**Figure 5:**
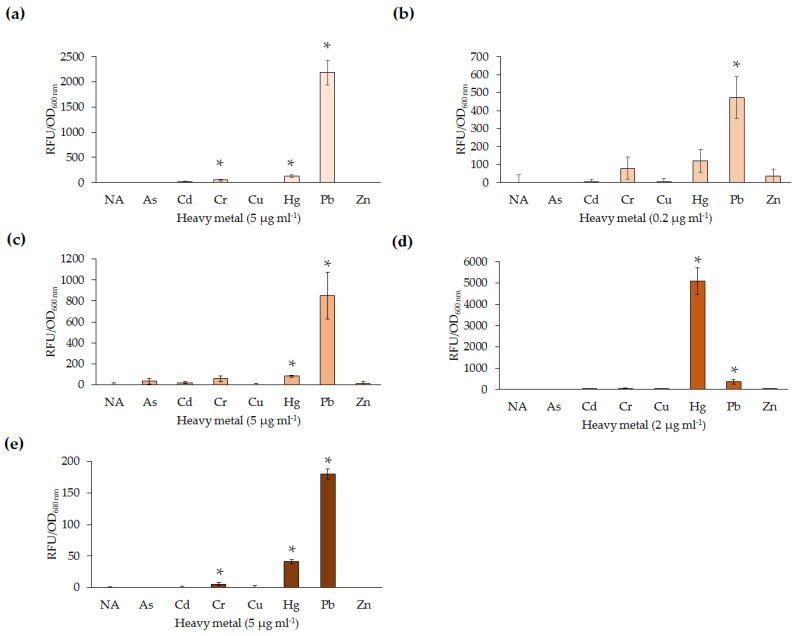
The five lead biosensors were exposed to a range of heavy metals at varying concentrations. The *p*-value (* *p* < 0.05) indicates a significant increase in RFU per OD_600 nm_ when compared to that of the uninduced sample (NA). (**a**) *E. coli* DH5α(pBB*pbrRgfp*) (5 µg·mL^−1^); (**b**) *P. aeruginosa* PAO1(pBB*pbrRgfp*) (0.2 µg·mL^−1^); (**c**) *S. oneidensis* MR-1(pBB*pbrRgfp*) (5 µg·mL^−1^); (**d**) *Enterobacter* sp. NCR3(pBB*pbrRgfp*) (2 µg·mL^−1^); and (**e**) *Enterobacter* sp. LCR17(pBB*pbrRgfp*) (5 µg·mL^−1^). Note: For *Enterobacter* sp. LCR17(pBB*pbrRgfp*) only one biological replicate is shown. NA: Not applicable; As: sodium arsenite; Cd: cadmium chloride; Cr: chromium oxide; Cu: copper chloride; Hg: mercury chloride; Pb: lead nitrate; and Zn: zinc chloride.

Previous studies have noted the lack of specificity of lead biosensors; therefore, the specificity of the five biosensors was investigated using a range of heavy metals. The most sensitive biosensor, *P. aeruginosa* PAO1(pBB*pbrRgfp*) was also the most specific (Figure 5b). At 0.2 µg·mL^−1^ of each heavy metal ion tested, *P. aeruginosa* PAO1(pBB*pbrRgfp*) was only able to detect Pb(II) at statistically significant levels following 4 h incubation. The other four lead biosensors detected Hg(II) at the same concentration as Pb(II). *Enterobacter* sp. NCR3(pBB*pbrRgfp*) was unexpectedly found to produce significantly higher expression levels in response to 1 µg·mL^−1^ of Hg(II), in comparison to Pb(II) (Figure 4d). Furthermore, *E. coli* DH5α(pBB*pbrRgfp*) and *Enterobacter* sp. LCR17(pBB*pbrRgfp*) also detected Cr(VI) at 5 µg·mL^−1^.

**Figure 6:**
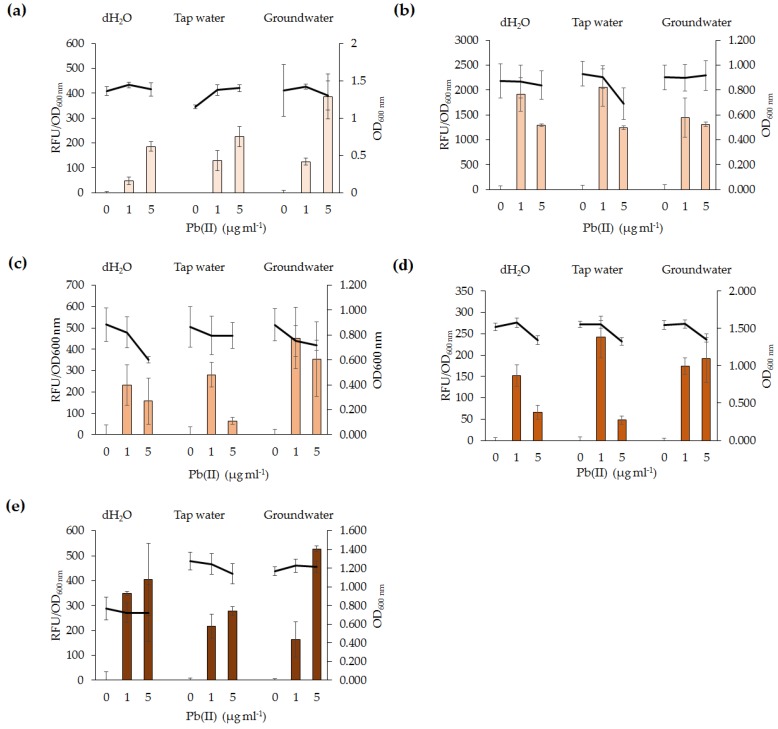
The lead biosensors were exposed to varying concentrations (0, 1, and 5 µg·mL^−1^) of Pb(II) in the following environmental samples; sterile dH_2_O, unsterilized tap water and unsterilized groundwater. (**a**) *E. coli* DH5α(pBB*pbrRgfp*); (**b**) *P. aeruginosa* PAO1(pBB*pbrRgfp*); (**c**) *S. oneidensis* MR-1(pBB*pbrRgfp*); (**d**) *Enterobacter* sp. NCR3(pBB*pbrRgfp*); and (**e**) *Enterobacter* sp. LCR17(pBB*pbrRgfp*).

To determine the effectiveness of the five lead biosensors to detect Pb(II) in environmental samples, tap water and groundwater, were spiked with 1 and 5 µg·mL^−1^ Pb(II). All five biosensors were able to detect 1 µg·mL^−1^ Pb(II), regardless of the water sample (Figure 6). With the exception of *S. oneidensis* MR-1(pBB*pbrRgfp*) the biosensors were able to detect 5 µg·mL^−1^ Pb(II), however, it was noted that an increase in Pb(II) concentration did not correlate with an in increase in RFU. No significant difference (*p* > 0.05) was observed when comparing growth (OD_600 nm_) of the cells exposed to 0, 1, or 5 µg·mL^−1^ Pb(II) of a given water sample except for *Enterobacter* sp. LCR17(pBB*pbrRgfp*). The reason for the observed difference in cell density between the control and tested samples is unclear.

## 4. Discussion

In this study, a lead biosensor construct (pBB*pbrRgfp*) was developed and introduced into several Gram-negative bacterial species, which were then exposed to a range of Pb(II) concentrations for various time periods. Like many other heavy metals, lead can also exist in a number of different forms, oxides and hydroxides, as well as oxyanion complexes that are released into the soil and groundwater. The most common form, Pb(II), was chosen for testing of the biosensors in addition to several other heavy metal ions to investigate the specificity of the developed biosensors.

The five lead biosensors developed in this study were able to detect between 0.2 and 1 µg·mL^−1^ Pb(II) following 4 h incubation. *P. aeruginosa* PAO1(pBB*pbrRgfp*) was deemed the most sensitive. This may be due to a higher expression of GFP in *Pseudomonas*, as a consequence of reduced transcriptional repression by PbrR. Upon exposure to Pb(II) for 10 h, the sensitivity of the *Pseudomonas* lead biosensor increased from 0.2 to 0.05 µg·mL^−1^ (data not shown). It can, thus, be proposed that, upon further incubation, the *Pseudomonas* lead biosensor could detect below 0.01 µg·mL^−1^, which is the WHO guideline level for Pb(II) in drinking water [7]. The limit of detection of previously reported lead biosensors varies significantly. For example, *A. eutrophus* luminescent lead biosensor detected ~331 µg·mL^−1^ Pb (compound not specified) [22]. In a separate study, *S. aureus* and *B. subtilis* luminescent biosensors (p*cadClucFF*) detected 0.01 µg·mL^−1^ Pb(CH_3_COO)_2_ after ~2 h exposure [19]. Some of the factors which can be attributed to differences in detection limits of lead biosensors developed include the genetic element used, the choice of the microorganism, the medium used, as well as the reporter gene. The growth phase of the biosensor culture as well as the concentration of the cells exposed to lead affect the limit of detection. Limitations to the limit of detection may include the culture reaching a stationary phase, which is largely influenced by the availability of nutrients. Nutrient availability influences the growth rate of the organism [19]. Furthermore, differences in copy number of the plasmid used may contribute to the sensitivity of lead detection and variability observed among different studies which have utilized a range of different plasmids. Therefore, future studies should utilize a high-copy number, broad-host range plasmid.

Slight variation of detection abilities (0.2–1 µg·mL^−1^) among the lead biosensors were further highlighted upon analysis of response times following exposure to Pb(II). Out of the five biosensors, *E. coli* DH5α(pBB*pbrRgfp*) and *Enterobacter* sp. LCR17(pBB*pbrRgfp*) responded quickest at 30 and 40 min, respectively. However, this may be attributed to differences in the growth rate of the microorganisms, as well as the different concentrations of Pb(II) used to test the response times. For example, *E. coli* DH5α(pBB*pbrRgfp*) was exposed to 5 µg·mL^−1^ whereas *P. aeruginosa* PAO1(pBB*pbrRgfp*) was exposed to 0.2 µg·mL^−1^. Response times are highly dependent on the concentration of the heavy metal used and the growth rate of the microorganism. Although a constant concentration could have been used, due to the variability of the lead biosensors’ response to Pb(II), the lowest detectable amount or above was chosen for testing the response times in this study. Previous studies have reported only induction time for lead biosensors tested [19,22] and as such, it would be beneficial to determine response times during future analyses.

All five lead biosensors developed could detect low levels of Pb(II) (0.2–1 µg·mL^−1^) following exposure for 30–100 min. Additionally, the biosensors were able to detect at least one heavy metal ion other than Pb(II), in most instances Hg(II). *E. coli* DH5α(pBB*pbrRgfp*) and *Enterobacter* sp. LCR17(pBB*pbrRgfp*) were also responsive to Cr(VI). Interestingly, the response of lead biosensors to Cr(VI) has not previously been reported. Similarly, reported Cr(VI) microbial biosensors were not tested for response to Pb(II) [29]. Nevertheless, non-specificity of the biosensor constructs was expected due to the similarity of heavy metal ions, as well as previous reports of non-specificity among lead biosensors [19]. For example, the *S. aureus* and *B. subtilis* luminescent lead biosensors detected Cd(II), Hg(II), Mn(II), Sb(III), Sn(II), and Zn(II) [19,20]. The differences in specificity between various microorganisms may be attributed to different methods of metal uptake into the cell. In contrast to non-specific *S. aureus* and *B. subtilis* lead biosensors, The *A. eutrophus* luminescent lead biosensor only detected Pb (compound not specified) [22]. This high level of specificity may be due to the medium used or the higher concentration of Pb (331 µg·mL^−1^) used for testing. Interestingly, *Enterobacter* sp. NCR3(pBB*pbrRgfp*) exhibited an elevated response to Hg(II) in comparison to the same concentration of Pb(II). As Hg(II) is generally more toxic than Pb(II), up-regulation of native Hg(II) resistance mechanisms may be responsible for increased uptake and binding of Hg(II) over Pb(II). In nature, the ability to sense a range of heavy metals in the surrounding environment may be hypothesized to provide microorganisms a competitive advantage, resulting in enhanced survival.

The potential of lead biosensors developed for in situ applications was assessed. All five lead biosensors detected 1 µg·mL^−1^ Pb(II) in tap water and groundwater. Testing of biosensors in environmental samples which have not been treated via sterilization or filtering is useful to determine the effects of complex environmental samples with resident microbial community. Our findings indicate that short-term exposure of the biosensors to contaminated water samples yield a response to Pb(II). Organic compounds and resident microbial communities in the water samples did not affect the functionality of the lead biosensors. The difference in absolute RFU values between fluorescence assays of the same bacterial species may be explained by the natural variability of the cultures. Overall, the lead biosensors are sensitive, rapid, and generally specific for Pb(II). As such, they could serve as monitoring tools for in situ ecotoxicological assessment of lead-polluted environmental samples. The findings in this study have highlighted variations among biosensors utilizing the same genetic element and, as such, differences between bacterial species should be addressed in future microbial biosensor studies. Future biosensor research should focus on utilizing organisms involved in bioremediation processes, such as immobilization or phytoremediation, and incorporating the biosensor genetic element into their chromosomes. It would also be beneficial for future biosensor studies to develop a more high-throughput method for analysis of microbial biosensors and for detection of Pb(II) in other environmental samples, such as wet weather effluent. This may be performed in parallel to analytical methods for comparison of accuracy and feasibility of the lead biosensors to detect Pb(II) in various environmental samples. 

## Figures and Tables

**Table 1:** List of strains and plasmids used in this study.

Strain/Plasmid	Relevant Characteristics	Reference/Source
**Strain**		
*Escherichia coli* DH5α	Standard cloning host; Nal^r^	Stratagene
*Escherichia coli* WM3064	DAP auxotroph; ∆*dapA*1341	[24]
*Pseudomonas aeruginosa* PAO1	Prototroph	[25]
*Shewanella oneidensis* MR-1	Prototroph, metal-reducing strain	[26,27]
*Enterobacter* sp. NCR3	Prototroph, wild-type *Enterobacter* sp.	[23]
*Enterobacter* sp. LCR17	Prototroph, wild-type *Enterobacter* sp.	Liu, W. (pers. comm.)
**Plasmid**		
pBBR1MCS-5	Broad host range cloning vector; Gm^r^	[28]
pCR2.1^®^-TOPO^®^ TA	Cloning vector; Ap^r^	Invitrogen^TM^
pTOPO*pbrRgfp*	pCR2.1^®^-TOPO^®^ TA containing *pbrR*, divergent P*_pbr_* promoter region and promoterless *gfpmut3b*; Ap^r^	This study
pBB*pbrRgfp*	pBBR1MCS-5 containing *pbrR*, P*_pbr_* divergent promoter region and promoterless *gfpmut3b* cloned into the *Eco*RI; Gm^r^	This study

Ap^r^; ampicillin resistance, DAP; diaminopimelic acid, Gm^r^; gentamicin resistance, Nal^r^; nalidixic acid resistance.
